# Evaluating the Potential of Ursolic Acid as Bioproduct for Cutaneous and Visceral Leishmaniasis

**DOI:** 10.3390/molecules25061394

**Published:** 2020-03-19

**Authors:** Pablo Bilbao-Ramos, Dolores R. Serrano, Helga Karina Ruiz Saldaña, Juan J. Torrado, Francisco Bolás-Fernández, María Auxiliadora Dea-Ayuela

**Affiliations:** 1 Departament of Microbiology and Parasitology, School of Pharmacy, Universidad Complutense de Madrid, Plaza Ramón y Cajal s/n, 28040 Madrid, Spain; pablobil15@yahoo.com (P.B.-R.); francisb@ucm.es (F.B.-F.); 2 Laboratorio de Parasitología y Entomología INLASA, Pasaje Rafael Zubieta #1889, (Lado Estado Mayor del ejército) Zona Miraflores, La Paz, Bolivia; 3 Departament of Pharmaceutics and Food Technology, School of Pharmacy, University Complutense, Avenida Complutense, 28040 Madrid, Spain; drserran@ucm.es (D.R.S.); helgakar@ucm.es (H.K.R.S.); torrado1@ucm.es (J.J.T.); 4 Institute of Industrial Pharmacy (IUFI), Plaza Ramon y Cajal, SN, 28040 Madrid, Spain; 5 Departament of Pharmacy, School of Health Science, Universidad CEU Cardenal Herrera, C/Ramón y Cajal s/n, 46115 Alfara del Patriarca (Valencia), Spain

**Keywords:** ursolic acid, visceral leishmaniasis, acute-infection, chronic-infection, cutaneous leishmaniasis, cytokines

## Abstract

Leishmaniasis affects around 12 million people worldwide and is estimated to cause the ninth-largest disease burden. There are three main forms of the disease, visceral (VL), cutaneous (CL), and mucocutaneous (MCL), leading to more than one million new cases every year and several thousand deaths. Current treatments based on chemically synthesized molecules are far from ideal. In this study, we have tested the in vitro and in vivo efficacy of ursolic acid (UA), a multifunctional triterpenoid with well-known antitumoral, antioxidant, and antimicrobial effects on different *Leishmania* strains. The in vitro antileishmanial activity against the intracellular forms was six and three-fold higher compared to extracellular forms of *L. amazonensis* and *L. infantum,* respectively. UA also showed to be a potent antileishmanial drug against both VL and CL manifestations of the disease in experimental models. UA parenterally administered at 5 mg/kg for seven days significantly reduced the parasite burden in liver and spleen not only in murine acute infection but also in a chronic-infection model against *L. infantum*. In addition, UA ointment (0.2%) topically administered for four weeks diminished (50%) lesion size progression in a chronic infection model of CL caused by *L. amazonensis,* which was much greater than the effect of UA formulated as an O/W emulsion. UA played a key role in the immunological response modulating the Th1 response. The exposure of *Leishmania-*infected macrophages to UA led to a significant different production in the cytokine levels depending on the *Leishmania* strain causing the infection. In conclusion, UA can be a promising therapy against both CL and VL.

## 1. Introduction

Leishmaniasis affects around 12 million people worldwide and is estimated to cause the ninth-largest disease burden [1]. There are three main forms of the disease, visceral (VL), cutaneous (CL) and mucocutaneous (MCL) [2]. Approximately 0.7 to 1.2 million new cases of CL, 0.2 to 0.4 million new VL cases, and 20,000 to 30,000 deaths occur annually. Around 310 million people are at risk of infection who live in one of the 98 endemic regions, most of them being developing countries [1,2]. CL causes ulcers on exposed parts of the body leading to permanent scars and disfigurement, stigma, and disability. Nevertheless, VL affects the vital organs in the body being the most severe form, which is fatal if left untreated [2]. Over twenty *Leishmania* spp. are known to transmit the disease to humans. According to the species and the differences in virulence, the infections may vary from simple cutaneous lesions to mucocutaneous ulcers or visceral diseases [3,4].

The chemotherapy of leishmaniasis has been based on the use of antimonial compounds, such as sodium stibogluconate and meglumine antimoniate, which are far from being the ideal treatments as they display a variable efficacy against VL and CL, cause severe side effects and are parenterally administered [5]. Currently, liposomal amphotericin B is the choice of therapy in VL. However, its high cost hampers its use in developing countries where conventional amphotericin B is still used in spite of causing severe nephrotoxicity [6]. Pentavalent antimonials are still the first-line drug but exhibit several limitations, including severe side effects, the need for daily parenteral administration and drug resistance [7]. Other medicines utilized against leishmaniasis are pentamidine and paromomycin, characterized by low cost and variable efficacy, and miltefosine, which is the only drug approved for oral administration but has the disadvantages of being teratogenic and leading to emerging resistances [8,9]. Many factors, including lack of efficacy, severe adverse effects, long and high cost therapies, and parenteral administration of most drugs, result in poor patient compliance and, at the same time, increases the risk of emerging resistant strains. Definitively, there is a clinical need for safer, more effective, and inexpensive treatments against all the leishmaniasis forms.

Currently, major research efforts are focused on discovering new natural compounds with antileishmanial properties [10]. Many active compounds with remarkable biological activities have been isolated from different plant species, such as flavonoids [11,12], anthocyanidins [13], coumarins [14], and triterpenoids [15].

Triterpenoids are polycyclic compounds derived from the linear hydrocarbon squalene and comprise one of the most promising groups of phytochemicals due to its multifunctionality in the treatment of a broad range of diseases [16,17,18,19,20,21]. For instance, oleanolic acid administered at low doses has hepatoprotective properties, whereas high doses can result in cholestasis and hepatotoxicity [22].

Ursolic Acid (UA) is a ubiquitous triterpenoid in the plant kingdom [23]. UA is a promising anticancer drug. Phase I clinical studies have already been performed in order to evaluate its toxicity and pharmacokinetic profile in healthy volunteers and patients with advanced solid tumors [24,25]. The intravenous infusion of encapsulated UA in liposomes was well tolerated, showing manageable toxicities with a maximum tolerated-dose of 98 mg/mL. Besides its antitumoral activity, UA possesses a wide variety of biological activities, such as antioxidant [26], hepatoprotective [22], hypoglycaemic [27], antibacterial [26], antiviral [28], antifungal [29] and antiprotozoal [18]. Its antiprotozoal efficacy has been tested in vitro against *Trypanosoma cruzi* [21], *Plasmodium falciparum* [22], and *Leishmania* spp [30].

Regarding the immunological effect of triterpenes, several studies have shown that they possess anti-inflammatory activity, mainly caused either by inhibiting enzymes involved in the production of eicosanoids, such as cyclooxygenases, or inhibiting the release of cytokines [31]. This anti-inflammatory effect is crucial against several infections, such as those caused by intracellular pathogens. In *Mycobacterium tuberculosis*, UA has shown the ability to inhibit the expression of certain inflammatory cytokines, such as TNF-α, IL-1β, IL-6, and TGF-β [32,33]. UA also has exhibited activity against other intracellular pathogens, such as *Toxoplasma gondii*, leading to an increased production of NO, ROS, IL-10, IL-12, granulocyte macrophage colony stimulating factor (GM-CSF) and interferon-β, while reducing the expression of IL-1β, IL-6, TNF-α and TGF-β in infected immune cells [34].

Several plant extracts from Brazil have shown in vitro activity against *L. amazonensis* and *L. braziliensis*. This activity has been attributed to the fraction of *Baccharis uncinellase* containing oleanolic and ursolic acids [35]. The same fraction has also exhibited in vivo efficacy in tegumentary leishmaniasis, resulting in a decreased skin parasitism and increased levels of IL-12 and IFN-γ, spreading the Th1 immune response [20]. In addition, the same plant extract was effective in vivo against *L. infantum* [36]. UA extracted from *Petiveria alliaceae* showed similar activity against *L. amazonensis* associated with programmed cell death of parasites and an increase in NO [37].

The aim of this study was to evaluate the in vitro activity of UA on promastigotes and intracellular amastigotes of different *Leishmania* species in order to select an adequate dose for in vivo efficacy studies. Moreover, topical formulations containing UA were developed, and its efficacy was tested in a chronic model of CL. The activity of parenterally administered UA was assessed on both an acute and chronic model of VL.

## 2. Results

### 2.1. Assessment of Topical Formulations and Quantification of UA

UA is a triterpenoid with negligible aqueous solubility. For this reason, it was compounded in semisolid formulations to facilitate its solubility and its topical administration. UA was readily dispersed using a mixture of glycerin: propylene glycol (1:1, w:w) that avoided drug agglomeration after mixing with the preformed emulsion or Orabase in the case of the cream or ointment, respectively. Both formulations exhibited a pH of 5.5, which is suitable for skin administration. UA was chemically stable over one month at 25 ± 2 °C. Consistency, color, and pH were kept unchanged over this period of time. UA concentration was determined using a linear regression calibration curve between 0.78 to 200μg/mL (y = 3.2423x + 0.4212; R^2^ = 0.9999).

### 2.2. In Vitro Activity against Leishmania spp. and Cytotoxicity against J774 Macrophages

Miltefosine showed greater in vitro activity on promastigotes than UA. However, UA exhibited an SI ranging from two to four, depending on the *Leishmania* spp., further in vitro investigations were performed against *Leishmania* amastigotes (Table 1).

The anti-leishmanial activity of UA against amastigotes showed a six and three-fold increase compared to extracellular forms of *L. amazonensis* and *L. infantum,* respectively (Table 2). Also, the selectivity index of UA was much greater (three and eight-fold higher depending on the strain) than miltefosine. The greater activity of UA observed in amastigotes rather than in promastigotes can be explained by the dual mode of action of this compound not only on the parasite cell but also stimulating the immunological response of the cell host, mainly increasing NO production in macrophages.

### 2.3. In Vivo Activity against L. infantum

#### 2.3.1. Efficacy in Acute Infection Model of VL in BALB/c

Supported by the in vitro leishmanicidal efficacy in both intracellular and extracellular forms, UA was also evaluated in vivo in a murine model of acute infection by *L. infantum*. The compound was administered by the intraperitoneal route at 5 mg/kg daily for seven consecutive days. The results are summarized in Table 3. UA showed a high parasite growth inhibition corresponding to 99.83% and 99.78% reduction in the number of parasites in spleen and liver, respectively, compared to the untreated group (*p* < 0.05). These results suggest a good UA bioavailability from the site of injection to the target organs. No evident signs of toxicity, like loss in weight or hair loss, were observed at the end of the experiment in any of the animals, which suggests that UA was well-tolerated by the infected mice at the administered dose.

#### 2.3.2. Efficacy in Chronic infection Hamster Model of VL

UA was also evaluated in vivo in a chronic infection model of VL developed in hamsters. Similarly, UA was administered by the intraperitoneal route at 5 mg/kg daily for seven consecutive days. The results are summarized in Table 4. In spite of the lack of evident signs of toxicity at the end of the treatment in any of the animals, UA was less effective against chronic than acute infection. At the end of the experiment, the mean weight in the treated group was higher (145.5 ± 13.4) than in the control group (126.6 ± 10.52), but there were no statistically significant differences. The natural compound showed a 58% and 79% reduction in the number of parasites in spleen and liver, respectively, compared to the untreated group.

#### 2.3.3. In Vivo Activity against *L. amazonensis* in an Experiment Model of CL

**Figure 1:**
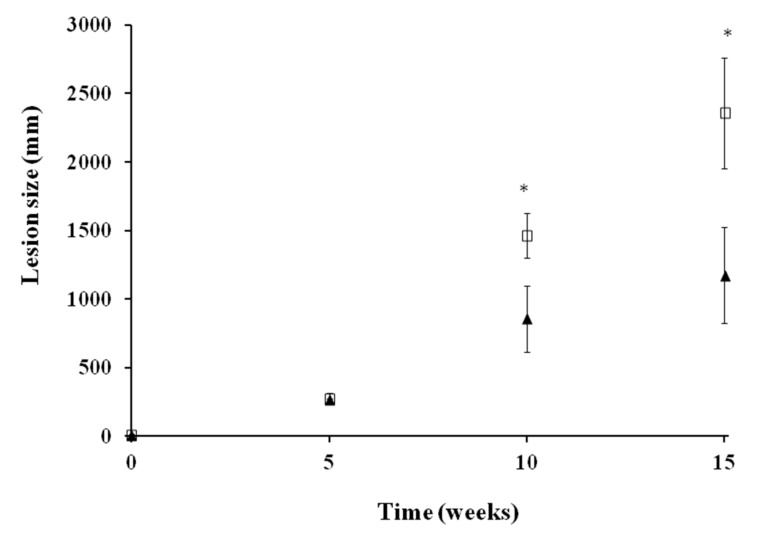
In vivo antileishmanial efficacy of UA ointment against *L. amazonensis* in a chronic infection model of cutaneous leishmaniasis (CL) in Syrian hamsters. Disease progression was monitored at week 0, 5, 10, and 15. After 35 days post-infection, UA ointment (0.2%) was administered once daily for 28 consecutive days in one of the groups. The other group received no treatment. Disease progression was monitored since the treatment was instaured at weeks 0, 5, 10, and 15. Lesion size (mean ± SD) is expressed as the difference between the diameter (mm) of the infected left hind paw and the non-infected right hind paw from each animal. Statistically significant differences (*p* < 0.05) are expressed as: * (U-Mann–Whitney). Key: UA ointment at 0.2% (-▲-); untreated group (-□-).

Prior to topical treatment, disease progression was similar in all animals, and no statistically significant differences were observed after 35 days post-infection (Figure 1). Daily topical administration of UA ointment (0.2%) for 28 days resulted in a significant (*p* < 0.05) 42% and 50.3% reduction in the lesion size compared to the untreated group at weeks 10 and 15. However, parasites were not completely eradicated after UA topical administration as inflammation increased at week 15.

**Figure 2:**
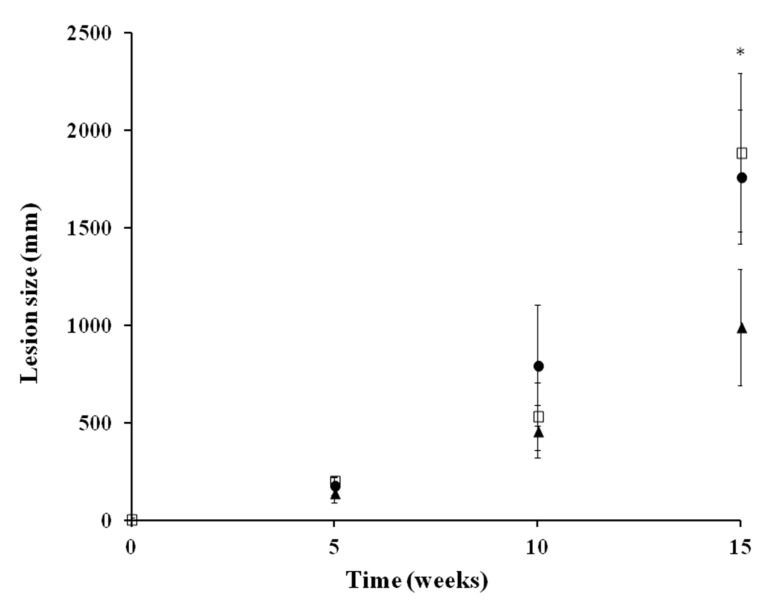
In vivo antileishmanial efficacy of UA ointment and cream against *L. amazonensis* in a chronic infection model of CL in hamsters. After 35 days post-infection, treatment with UA was topically administered once daily for 28 consecutive days. The first group received UA ointment (0.5%). The second group received UA cream (0.5%), and the third group was left untreated. Lesion size (mean ± SD) is expressed as the difference between the diameter (mm) of the infected left hind paw and the non-infected right hind paw from each animal. Statistically significant differences (*p* < 0.05) are expressed as: * (U-Mann–Whitney). Key: UA ointment at 0.5% (-▲-); UA cream at 0.5% (-●-); untreated group (-□-).

In a subsequent study, a higher dose of UA (0.5%) was tested (Figure 2). Daily topical administration of either UA ointment (0.5%) or UA cream (0.5%) for 28 days did not result in a significant reduction in the lesion size compared to the untreated group at week 10, although a significant reduction (47.54%) in inflammation size was achieved at week 15 after the administration of UA ointment.

### 2.4. Production of Cytokines

The efficacy of UA was higher on the intracellular amastigotes than in the promastigotes. This can be linked with an immunological reaction of the macrophages. For this reason, the effect of UA on the production of cytokines in different types of cell cultures was evaluated: (i) naïve splenocytes from mouse spleen and (ii) infected macrophages with *L. infantum* and *L. amazonensis*. On the splenocytes, out of the 17 studied cytokines, the following ones showed a significant increase in the presence of UA: GM-CSF, IFN-γ, IL-4, IL-6, IL-9, IL-10, and decrease of RANTES (Table 5). Nevertheless, IL4, IL6, and IL-10 decreased at higher concentrations of UA. In contrast, higher UA concentrations led to an increase in IFN-γ, which can make the eradication of intracellular forms of *Leishmania* favorable, opposite to the decrease in IL-10.

It is also key to investigate the production of cytokines on infected macrophages and not only just on lymphocytes. The exposure of *Leishmania-*infected macrophages to UA led to a significant different production in the cytokines levels depending on the *Leishmania* strain causing the infection. Except for the production of TNF-α that was exacerbated in both cases, several differences were observed in the levels of: GM-CSF, IFN-γ, IL-1b, IL-2, IL-6, IL-10, and MCP-1.

In *L. infantum*-infected macrophages, the production of GM-CSF, IL-6, and IL-10 was augmented compared to the untreated control while the levels of IFN-γ, MCP-1, and IL-1b were diminished significantly (*p* < 0.05) when *L. amazonensis* infected macrophages were exposed to UA (Table 6).

## 3. Discussion

In vitro antileishmanial activity of UA has been previously reported by other authors [35,36,37]. However, this is one of the first works that reports the in vivo leishmanicidal activity of UA against both VL and CL in different states of the disease. There is a wide variability in the reported in vitro efficacy against *Leishmania* spp. Many factors can contribute to this variability, but the natural source from which UA is extracted plays a key role in the final activity of this metabolite.

Tan et al. extracted UA from *Salvia cilicica* roots, which exhibited a very high in vitro activity in the nanomolar range against *L. donovani* and *L. major* promastigotes (IC_50_ equal to 91 and 51.3 nM, respectively) and amastigotes (IC_50_ equal to 12.7 and 7.0 nM) [38]. Moulisha et al. obtained UA from *Terminalia arjuna* displaying an IC_50_ of 3.51 μg/mL against *L. donovani* promastigotes [39]. Odonne et al. extracted UA from *Pseudelephantopus spicatus* (peruvian plant), which exhibited a high in vitro activity (IC_50_ equal to 0.20 µM) against *L. amazonensis* [40].

On the contrary, UA extracted from *Miconialang sdorffii* exhibited a very low antileishmanial activity against *L. amazonensis* promastigotes (IC_50_ de 350 μM) [41]. Chemically synthesized UA showed an IC_50_ of 20 and > 25 μM against *L. amazonensis* and *L. infantum* promastigotes, respectively [30].

This variable efficacy is one of the major problems of natural compounds. Extraction and purification processes should also be standardized in order to obtain a compound with reproducible characteristics, making its further use in clinical studies easier. For this reason, chemical synthesized UA (purchased by Sigma-Aldrich) was used to perform the in vitro and in vivo assays.

The higher leishmanicidal activity of UA against intracellular compared to extracellular forms has also been reported by other authors [39]. This fact may be related to its ability to modulate the macrophage nitric oxide production, which would enhance the death of the intracellular parasites [42].

Parenteral administration of UA at 5 mg/kg for seven consecutive days has shown to significantly reduce parasite growth in liver and spleen in both acute and chronic infection models of VL. In the acute-infection mouse model, although there is parasite growth in the liver and spleen, the infection is usually resolved by a Th_1_ dependent granulomatous response after multiple weeks [43]. However, VL developed by hamsters closely resembles human VL. It is characterized by a relentless growth of parasites in the spleen, liver, and bone marrow, being more complicated to eradicate and usually ends up killing the animal [43]. It has been reported that UA can improve the Th1 immune response in infected mice, which could explain its potent antileishmanial activity [20]. In this sense, the reduction in parasite burden not only in the acute-infection model but also in the chronic model makes UA a promising drug for VL therapy.

Results obtained after UA topical administration against CL were also encouraging suggesting that: (i) higher doses than 0.5% topically administered can lead to an altered immune-reaction in the cutaneous infected lesion, (ii) UA ointment formulation has a better permeability across the skin than UA cream, and (iii) a successful therapy against CL could be achieved if prolonged topical treatments using UA ointment (0.2%) are applied. Despite the fact that creams combine properties, such as lubrication, occlusion, and hydration, that lead to higher patient compliance and better suitability for cutaneous lesions (being especially useful in intertriginous areas where ointments might not be used because they may cause maceration or folliculitis [44,45]), ointments can be more convenient when an occlusive effect is required to enhance drug permeation [45].

The immune response associated with the protection against a *Leishmania* infection is attributed to the development of a Th1-type immunity characterized by the production of cytokines, such as IFN-γ, TNF-α, IL-12, and GM-CSF, which can lead to the activation of macrophages and production of nitric oxide (NO).

Passero et al. demonstrated that the production of cytokines in an in vivo model of *L. infantum*-infected hamsters was characterized by an increase in the IL-10 levels at all the doses tested of UA and a decrease in the IFN-γ at higher doses [35]. Our in vitro results correlate with those observed by Passero et al. In the latter study, it was also remarked that even though the parasite load were reduced by 96%, the complete eradication of the parasites was not achieved, which can be related to an immunoregulator effect of the parasite on the macrophages [36].

Other studies have shown that the antileishmanial activity of UA depends on two factors: the parasite strain as well as the interaction with the immune system. The production of IFN-γ in infected macrophages seems to be more regulated by the presence of the parasites rather than the concentration of UA. This can explain the lack of efficacy of UA in the chronic models of VL [46].

Other studies have shown that GM-CSF has a beneficial output in the disease progression, especially in CL, because it triggers the activation of macrophages to kill *Leishmania* [47,48]. GM-CSF can improve healing and scarring of cutaneous lesions caused by CL combined con different treatments, such as glucantime or miltefosine [48,49]. In our study, an increase in the GM-CSF levels was observed on lymphocytes independently of the dose of the UA tested. However, this increase was only noticeable in those macrophages infected by *L. infantum* along with higher levels of TNF-α. This can explain the higher activity of UA observed against amastigotes compared to promastigotes.

## 4. Materials and Methods

### 4.1. Chemical Reagents

All chemicals used, unless otherwise stated, were obtained from Sigma-Aldrich (Madrid, Spain) or Panreac S.A. (Barcelona, Spain) and used without further purification.

### 4.2. Preparation of UA Topical Formulations

UA cream consisted of an aqueous phase with tween 80 (6%) dispersed in water and an oil phase constituted by white wax (1%) and cetyl alcohol (15%) melted at 60⁰. The aqueous phase was heated at 60⁰ and then was added onto the oil phase at the same temperature. Continuous stirring was required until the cream reached the desired consistency at room temperature. Finally, UA (0.5%) dispersed in a mixture (1:1) of glycerin:propylene glycol (12%) was added.

UA ointment was prepared by dispersing the UA (0.2 or 0.5%) in a mixture (2:1) of glycerin:propylene glycol (5%), which, in a second step, was mixed with Orabase^®^ (Fagron, Madrid, Spain), a plasticized hydrocarbon gel composed of gelatin, pectin, and sodium carboxymethyl cellulose.

### 4.3. Quantification of UA in the Topical Formulations

UA concentration was assessed by HPLC. Formulations (50 mg) were weighted and dissolved with methanol (100 mL). Samples were centrifuged at 3000× *g* for 10 min, and supernatant was collected and injected in the HPLC. HPLC was equipped with a Jasco PU-1580 pump, a Jasco AS-2050 Plus autosampler, and a Jasco UV-1575UV–visible detector (Jasco, Madrid, Spain). Integration of the peaks was performed with the program Borwin 1.5 for PC (JMBS Developments, Tokyo, Japan). The samples (100 µL) were chromatographed over a ThermoHypersil BDS C18 reverse-phase column (Thermo Fisher Scientific, Madrid, Spain) (200 × 4.6 mm, 5 μm) at a flow rate of 1 mL/min. Elution was carried out isocratically with a mobile phase that consisted of a methanol:water (95:5, *v*/*v*) mixture filtered through a 0.45 µm hydrophilic polypropylene filter membrane (GH polypro, Pall Corp., DeLand, FL, USA). UA was detected at a wavelength of 210 nm, and its retention time was 5 min.

### 4.4. Parasites and Culture Procedure

The following species of *Leishmania* were used: an autochthonous isolate of *L. infantum* (MCAN/ES/96/BCN150) kindly given by Prof. José M. Requena (Universidad Autónoma de Madrid, Madrid, Spain); *L. braziliensis* 2903, *L. amazonensis* (MHOM/Br/79/Maria), and *L. guyanensis* 141/93 were kindly provided by Prof. Alfredo Toraño (Instituto de Salud Carlos III, Madrid, Spain). Promastigotes were cultured in Schneider’s Insect Medium supplemented with 10% heat-inactivated Foetal Bovine Serum (FBS) and 1000 U/L of penicillin plus 100 mg/L of streptomycin in 25 mL culture flasks at 26 °C.

#### 4.4.1. In Vitro Promastigote Susceptibility Assay

The assay was performed as previously described [50]. Briefly, log-phase promastigotes (2.5 × 10^5^ parasites/well) were cultured in 96-well plastic plates. Compounds (UA and miltefosine) were dissolved in dimethylsulfoxide (DMSO) and different concentrations of each (100, 50, 25, 12.5, 6.25 3.12, 1.56, and 0.78 μg/mL) were carried out up to 200 µL final volume. Growth control and signal-to-noise were also included. The final solvent (DMSO) concentrations never exceeded 0.5% (*v*/*v*) warranting no effect on parasite proliferation or morphology. After 48 h at 26 °C, 20 μL of a 2.5 mM resazurin (Sigma-Aldrich, Madrid, Spain) solution was added to each well, and the plates were returned to the incubator for another 3 h. The Relative Fluorescence Units (RFU) (535–590 nm excitation–emission wavelength) was determined in a fluorometer (Infinite 200Tecan i-Control, Barcelona, Spain). Growth inhibition (GI) was calculated by the following equation:

(1) $$GI\;\left(\%\right)=100-\frac{RFU\;treated\;wells-RFU\;signal\;to\;noise}{RFU\;untreated\;wells-RFU\;signal\;to\;noise}\;\times \;100$$

All tests were carried out in triplicate. Miltefosine was used as the reference drug and was evaluated under the same conditions. The efficacy of each compound was estimated by calculating the IC_50_ (concentration of the compound that produced a 50% reduction in parasites) using a multinomial probit analysis incorporated in SPSS software v21.0 (IBM, Madrid, Spain).

#### 4.4.2. In Vitro Intracellular Amastigote Susceptibility Assay

The assay was carried out as previously described [51]. Briefly, 5 × 10^4^ J774 macrophages and stationary promastigotes in a 1:10 ratio were seeded in each well of a microtiter plate, suspended in 200 μL of culture medium and incubated for 24 h at 33 °C in 5% CO_2_ chamber. After this first incubation, the temperature was increased up to 37 °C for another 24 h. Thereafter, cells were washed several times in culture medium by centrifugation at 1500 rpm for 5 min in order to remove free non-internalized promastigotes. Finally, the supernatant was replaced by 200 μL/well of culture medium containing 2-fold serial dilutions of the test compounds (50, 25, 12.5, 6.25 3.12, 1.56, 0.78, and 0.39 μg/mL). Growth control and signal-to-noise were also included. All tests were carried out in a triplicate assay. Following incubation for 48 h at 37 °C, 5% CO_2_, the culture medium was replaced by 200 μL/well of the lysis solution (RPMI-1640 with 0.048% HEPES and 0.006% SDS) and incubated at room temperature for 20 min. Thereafter, the plates were centrifuged at 3500 rpm for 5 min, and the lysis solution was replaced by 200 μL/well of Schneider´s insect medium. The culture plates were then incubated at 26 °C for another 4 days to allow the transformation of viable amastigotes into promastigotes and proliferation. Afterward, 20 μL/well of 2.5 mM resazurin was added and incubated for another 3 h. Finally, fluorescence emission was measured, and IC_50_ was estimated as described above. All tests were carried out in triplicate. Miltefosine was used as the reference drug and was evaluated at the same conditions.

#### 4.4.3. Cytotoxicity Assay on Macrophages

The assay was carried out as previously described [52]. J774 macrophages cell lines were seeded (5 × 10^4^ cells/well) in 96-well flat-bottom microplates with 100 μL of RPMI 1640 medium. The cells were allowed to attach for 24 h at 37 °C, 5% CO_2_, and the medium was replaced by different concentrations of the compounds in 200 μL of medium and exposed for another 24 h. Growth controls and signal-to-noise were also included. Afterward, a volume of 20 μL of the 2.5 mM resazurin solution was added, and plates were returned to the incubator for another 3 h to evaluate cell viability. The reduction of resazurin was determined by fluorometry as in the promastigote assay. Each concentration was assayed three times. Cytotoxicity effect of compounds was defined as the 50% reduction of cell viability of treated culture cells with respect to untreated culture (CC_50_).

### 4.5. In Vivo Studies

Male BALB/c mice of 20–25 g body weight and male golden hamsters (*Mesocricetus auratus*) of 60–70 g were obtained from Harlan Interfauna Ibérica S.A. (Barcelona, Spain). Animals were allocated in plastic cages in a 12 h dark–light cycle animal with controlled temperature (25 °C) and humidity (70%). Water and food were unrestricted throughout the study. All the animals were handled according to the European Union legislation Directive 2010/63/EU and Spanish law Real Decreto 53/2013 on the protection of animals used for scientific purposes with an ethical committee code CEXAN170415.

#### 4.5.1. Acute Infection Model of VL in BALB/c

Each mouse was infected by an intracardiac route with 1 × 10^7^promastigotes of *L. infantum* according to a model previously described by Dea-Ayuela et al. [53]. Mice were randomly split into two groups each of six animals. Treatment started on day thirty-five post-infection and lasted for seven consecutive days. One group received UA at 5 mg/kg once daily intraperitoneally administered in 0.1 mL of saline solution. The other group was left untreated (control group). One week after the last treatment, all mice were sacrificed, and parasite burden in liver and spleen was estimated by the limit dilution assay describe elsewhere [54]. Briefly, spleen (0.15 g) and liver (0.5 g) were homogenized in a 5 mL of PBS–50 mM glucose–2 mM ethylene diamine tetraacetic acid (EDTA) solution at 4 °C using a sterilized steel stainless tissue grinder. Cell debris was removed by passage through a glass wool column. The suspension obtained was centrifuged at 1500 rpm for 15 min at 4 °C. Afterward, the supernatants were discarded, and the pellets were collected and resuspended in Schneider medium supplemented as described above, and then, 200 µL of this suspension were transferred to the first well of a 96-well microtiter plate containing medium supplemented with antibiotics, as described above. Serial dilutions were repeated, transferring 100 µL from the previous well to the next one and adding 100 µL of Schneider medium. After incubation at 26 °C for 7 days, microplates were examined using an inverted microscope (Olympus, model IM) at a magnification of 40X, and the presence or absence of promastigotes in each well was recorded. The final titer was the last dilution for which the well contained at least one parasite [54].

#### 4.5.2. Chronic Infection Model of VL in Golden Hamster

Chronic infection in Golden hamster was developed, as previously described by Dea-Ayuela et al. [55]. Each hamster was infected by an intracardiac route with 1 × 10^7^ promastigotes of *L. infantum*. Hamsters were randomly split into two groups of six animals. Treatment started on day thirty-five post-infection and lasted for seven consecutive days. One group received UA at 5 mg/kg once daily intraperitoneally administered in 0.1 mL of saline solution. The other group was left untreated (control group). Parasite burden in liver and spleen was estimated by the limit dilution assay described above [54].

#### 4.5.3. Chronic Infection Model of CL in Golden Hamster

This experiment was carried out as described by Morais-Teixeira et al., with some modifications [56]. Hamsters were randomly split into two groups of six animals. A suspension (25 µL) containing 1 × 10^7^ stationary promastigotes of *L. amazonensis* in Schneider medium was injected subcutaneously in the footpad of the left hind paw at day 0. Right hind paw was used as a negative control (no infection). After 5 weeks post-infection, chronic CL was developed, and topical treatment with UA started. In a first experiment, UA ointment (0.2%) was administered once daily for 4 consecutive weeks. The other group received no treatment. Disease progression was monitored at weeks 0, 5, 10, and 15 using a Vernier caliper to measure footpad size. In a second experiment, hamsters were randomly split into three groups of six animals. One group was treated with UA ointment (0.5%), and the other group was tread with UA cream (0.5%). As in the first experiment, the third group received no treatment (control group).

### 4.6. Effect of UA in the Cytokines Production in Macrophages Infected with Leishmania In Vitro

In vitro *Leishmania* infection was carried out as described previously. After the incubation with 200 μL/well of culture medium containing UA at the IC_50_ concentration previously determined for each of the species (*L. infantum* and *L. amazonensis*) for 48 h the supernatants were collected and stored at −80 °C for cytokine quantification.

#### 4.6.1. Splenocytes Culture and Evaluation of Cytokines Production

Cell suspension of splenocytes was prepared from the spleen of non-infected BALB/c mice and then was rinsed with RPMI 1640 medium three times. Then, 5 mL of the cell suspension was added to 1 mL of ammonium chloride 0.8% to isolate the splenocytes, and the suspension was again washed three times with RPMI 1640 medium. The splenocytes, 2.5 × 10^9^ cells/L, were cultured in RPMI 1640 medium containing 10% fetal bovine serum and incubated at 37 °C for 24 h. After incubation for 24 h, the supernatants were collected and stored at −80 °C for cytokine quantification.

#### 4.6.2. Analysis of Cytokines

The analyses were carried out by using the Quantibody^®^ array (RayBiotech, Madrid, Spain), a multiplexed sandwich ELISA-based quantitative array platform [57]. Nineteen different markers were analyzed: GM-CSF, IFNγ, IL-1a, IL-1β, IL-2, IL-3, IL-4, IL-5, IL-6, IL-9, IL-10, IL-12, IL-13, IL-17, KC, MCP-1, M-CSF, RANTES, VEGF. A pair of cytokine specific antibodies was used for detection. A capture antibody was first bound to the glass surface. After incubation with the sample, the target cytokine was trapped on the solid surface. A second biotin-labeled detection antibody was added, which recognized a different epitope of the target cytokine. The cytokine-antibody-biotin complex was visualized through the addition of the streptavidin-conjugated Cy3 equivalent dye, using a laser scanner (GenePix^®^ 4000B Scanner, UCM Facilities, Madrid, Spain).

A sandwich ELISA kit was used to determine the concentrations of TNF-α according the manufacturer’s instructions. Briefly, standards and samples (50 µL) were diluted with 50 µL of commercial diluting solution in triplicate and incubated for 2 h at 37 °C. Plates were washed five times, and 100 µL of conjugated anti-TNF-α was added and incubated for 1 h at 37 °C. Finally, 100 µL of TMB was added as substrate and incubated for 30 min in dark at room temperature. The reaction was stopped with 3M H_2_SO_4,_ and the optical densities (OD) were read at 450 nm.

### 4.7. Statistics

#### 4.7.1. Statistical and Data Analysis for In Vitro Assays

The efficacy against the parasite (IC_50_) and cytotoxicity effect (CC_50_) of compounds were calculated from Probit analysis using SPSS v21.0 software (IBM, Madrid, Spain) Finally, selective index (SI) was calculated defined as the ratio between CC_50_/IC_50._

The analysis of cytokines in vitro was performed by Tukey‘s HSD test using the statistical program IBM^®^ SPSS v21.0 (IBM, Madrid, Spain)_._

#### 4.7.2. Statistical and Data Analysis for in Vivo Assays

The group mean, standard deviation, standard error, and the differences were compared by the Mann–Whitney U nonparametric test, using the statistical programs of Microsoft Excel 2011^®^ (Microsoft, Washington, DC, USA) and IBM^®^ SPSS v21.0. Statistical significance was set at a *p*-value < 0.05.

## 5. Conclusions

Currently, plant-based therapies have shown great potential in the treatment of leishmaniasis. However, it is challenging to find a compound that possesses efficacy not only against cutaneous but also against visceral leishmaniasis. In this work, we have demonstrated that UA, a ubiquitous triterpenoid in nature, has a potent antileishmanial efficacy against both VL and CL, having a direct effect on the immunological response of the host. UA parenterally administered at 5 mg/kg significantly reduced parasite growth in liver and spleen not only in acute-infection but also in a chronic infection model. In addition, UA ointment (0.2%) topically administered could constitute a promising therapy against CL being able to penetrate across the skin and to diminish disease progression. In conclusion, formulating UA with suitable excipient can lead to promising treatments for cutaneous and visceral leishmaniasis.

## Figures and Tables

**Table 1:** In vitro leishmanicidal activity of ursolic acid (UA) on *Leishmania* promastigotes and cytotoxicity on J774 macrophages. Miltefosine was used as the reference drug.

Compound	IC_50_ (μg/mL) ^a^								CC_50_ (μg/mL) ^c^
	*Leishmania* *brazilensis*	SI ^b^	*Leishmania* *guyanensis*	SI ^b^	*Leishmania* *amazonensis*	SI ^b^	*Leishmania* *infantum*	SI ^b^	J774 macrophages
UA	17.0 ± 0.92	3.2	28.0 ± 0.09	2	14.1 ± 0.14	4	20.9 ± 1.40	2.7	55.8 ± 3.98
Miltefosine	7.16 ± 0.22	7.9	6.85 ± 0.35	8.2	12.44 ± 0.49	4.6	7.19 ± 0.60	7.9	57.1 ± 4.6

^a^ IC50, concentration of the compound that produced a 50% reduction in parasites; SD: standard deviation. ^b^ Selectivity index, SI = CC50/IC50. ^c^ CC50, concentration of the compound that produced a 50% reduction of cell viability in treated culture cells with respect to untreated ones.

**Table 2:** In vitro leishmanicidal activity of UA on *Leishmania* intracellular amastigotes. Miltefosine was used as the reference drug.

Compound	IC_50_ (μg/mL) ^a^			
	*Leishmania amazonensis*	SI ^b^	*Leishmania infantum*	SI ^b^
UA	2.24 ± 0.16	24.9	6.7 ± 0.6	8.3
Miltefosine	20.09 ± 1.47	2.8	23.7 ± 1.78	2.5

^a^ IC50, concentration of the compound that produced a 50% reduction in parasites; SD: standard deviation. ^b^ Selectivity index, SI = CC50/IC50.

**Table 3:** In vivo antileishmanial efficacy of UA against *L. infantum* in an acute infection model of visceral leishmaniasis (VL) in BALB/c. Parasite burden of the control (untreated) group and treated group with UA parenterally administered at 5 mg/kg daily for seven consecutive days. Results are expressed as mean parasite number ± standard deviation in spleen and liver per milligram.

Group	Parasite Burden (Amastigotes/mg Organ (× 10^3^)	
	Spleen	Liver
Treated group (with UA)	0.18 ± 0.02 *	0.06 ± 0.01 *
Untreated group (control)	111.84 ± 2.5	27.96 ± 7.8

* Treated control group vs. untreated show significant differences with *p*-value < 0.05 determined by Mann–Whitney U test.

**Table 4:** In vivo anti-leishmanial efficacy of UA against *L. infantum* in chronic infection model of VL in Syrian golden hamsters. Parasite burden of the control (untreated) group and treated group with UA parenterally administered at 5 mg/kg daily for seven consecutive days is expressed as mean parasite number ± standard deviation in spleen and liver per milligram.

Group	Parasite Burden (Amastigotes/mg Organ (×10^6^)	
	Spleen	Liver
Treated group (with UA)	9.75 ± 4.73	4.0 ± 2.88+*
Untreated group (control)	16.78 ± 5.30	19.04 ± 7.60

Treated control group vs. untreated show significant differences with *p*-value < 0.05 determined by the Mann–Whitney U test.

**Table 5:** Quantification of cytokine production of splenocytes untreated and treated with UA tested at the IC_50_ concentration corresponding to *L. amazonensis* and *L. infantum*.

Cytokines (pg/mL)	Splenocytes Mice BALBc		
	Untreated Group	Ursolic Acid (2.24 µg/mL)	Ursolic Acid (6.7 µg/mL)
GM-CSF	11.6 ± (3)	60.0 ± (10) *	100.7 ± (20) *
IFN-γ	0	44.4 ± (4.2) *	223.4 ± (87.7) **
IL-4	3.5 ± (0.9)	9.3 ± (1.4) *	6.4 ± (1.1)
IL-6	42.8 ± (5.6)	103.1 ± (11) *	50.2 ± (7.9)
IL-9	153.1 ± (20.5)	216.3 ± (9.6)	232.0 ± (25.5) *
IL-10	72.2 ± (17.6)	234.1 ± (36.2) *	135.8 ± (33.3) *
RANTES	16.2 ± (0.9)	1.6 ± (0.3) **	0 **

Statistically significant differences are expressed as * *p*-value < 0.05 and ** *p*-value < 0.01 (Tukey’s HSD-test).

**Table 6:** Quantification of cytokine production in *Leishmania*-infected macrophages untreated and treated with UA tested at IC_50_ values, respectively, for each parasite strain.

Cytokines (pg/mL)	*L. infantum/macrophages*		*L. amazonensis/macrophages*	
	Untreated Mean (ESM)	Ursolic Acid Mean (ESM)	Untreated Mean (ESM)	Ursolic Acid Mean (ESM)
GM-CSF	23.2 ± (4.4)	67.3 ± (2.8) *	49.8 ± (6.9)	44.8 ± (0.9)
IFN-γ	90.2 ± (125.9)	86.9 ± (9.8)	139.2 ± (20.2)	43.5 ± (26.8) *
IL-1b	23.4 ± (8.5)	24.5 ± (4.9)	42.3 ± (5.8)	20 ± (1.3) *
IL-2	0	3.9 ± (1.1) *	3 ± (0.4)	1.4 ± (0.4) *
IL-6	53.4 ± (11.4)	97.8 ± (7.2) *	71 ± (9.5)	66.8 ± (8.5)
TNF-α	0	22.72 ± (3.9)*	0	40.22 ± (6.9)*
IL-10	146.4 ± (10.8)	258.9 ± (14.6) *	197.1 ± (7.9)	184.1 ± (37.6)
MCP-1	33 ± (4.0)	50.3 ± (16.4)	147.9 ± (19.5)	37.1 ± (2.2) *

*ESM: Error Standard Mean;* Statistically significant differences (*p*-value < 0.05) are expressed as: * (Tukey´s HSD-test).
